# Supplementary material for: SARS-CoV-2 RNAemia and proteomic trajectories inform prognostication in COVID-19 patients admitted to intensive care
Source: Nat Commun. 2021 Jun 7;12:3406. doi: 10.1038/s41467-021-23494-1 (PMC8184784; doi:10.1038/s41467-021-23494-1)
Supplement: Supplementary file 1 — Supplementary Information [file 41467_2021_23494_MOESM1_ESM.pdf]

Supplementary Information

SARS-CoV-2 RNAemia and proteomic trajectories inform prognostication in COVID-19 patients admitted to intensive care

Clemens Gutmann[1*], Kaloyan Takov[1*], Sean A. Burnap[1*], Bhawana Singh[1*], Hashim Ali[1*], Konstantinos Theofilatos[1], Ella Reed[1], Maria Hasman[1], Adam Nabeebaccus[1,2], Matthew Fish,[3,4] Mark J.W. McPhail[2,5,6], Kevin O'Gallagher[1,2], Lukas E. Schmidt[1], Christian Cassel[1], Marieke Rienks[1], Xiaoke Yin[1], Georg Auzinger[2], Salvatore Napoli[5], Salma F. Mujib[6], Francesca Trovato[2,5,6], Barnaby Sanderson[4], Blair Merrick[7], Umar Niazi[8], Mansoor Saqi[8], Konstantina Dimitrakopoulou[8], Rafael Fernández-Leiro[9], Silke Braun[10], Romy Kronstein-Wiedemann[11], Katie J. Doores[3], Jonathan D. Edgeworth[3,7], Ajay M. Shah[1], Stefan R. Bornstein[12,13], Torsten Tonn[11,14], Adrian C. Hayday[3,15], Mauro Giacca[1], Manu Shankar-Hari[3,4,#], Manuel Mayr[1,12,#].

[1] King's College London British Heart Foundation Centre, School of Cardiovascular Medicine and Sciences, London, UK.

[2] King's College Hospital NHS Foundation Trust, London, UK.

[3] Department of Infectious Diseases, School of Immunology and Microbial Sciences, King's College London, London, UK.

[4] Department of Intensive Care Medicine, Guy's and St Thomas' NHS Foundation Trust, London, UK.

[5] Department of Inflammation Biology, School of Immunology and Microbial Sciences, Faculty of Life Sciences and Medicine, King's College London, London, UK.

[6] Institute of Liver Studies, King’s College Hospital, London, UK

[7] Clinical Infection and Diagnostics Research group, Department of Infection, Guy’s and St Thomas’ NHS Foundation Trust, London, UK

[8] NIHR Biomedical Research Centre, Guy's and St Thomas' NHS Foundation Trust and King's College London, London, UK

[9] Structural Biology Programme, Spanish National Cancer Research Centre (CNIO), Melchor Fernández Almagro 3, 28029 Madrid, Spain.

[10] Medical Clinic I, University Hospital Carl Gustav Carus, Technical University Dresden, Dresden, Germany

[11] Experimental Transfusion Medicine, Faculty of Medicine Carl Gustav Carus, Technical University Dresden, Dresden, Germany

[12] Department of Internal Medicine III, University Hospital Carl Gustav Carus, Technical University Dresden, Dresden, Germany

[13] Department of Diabetes, School of Life Course Science and Medicine, King's College London, London, UK

[14] Institute for Transfusion Medicine, German Red Cross Blood Donation Service North East, Dresden, Germany

[15] The Francis Crick Institute, London, UK

* These authors contributed equally.

[#] **To whom correspondence should be addressed:**

Prof. Manu Shankar-Hari; manu.shankar-hari@kcl.ac.uk or Prof. Manuel Mayr; King's British Heart Foundation Centre, King's College London, 125 Coldharbour Lane, London, SE59NU UK; Phone: +44(0)2078485446; +44(0)2078485298; manuel.mayr@kcl.ac.uk.

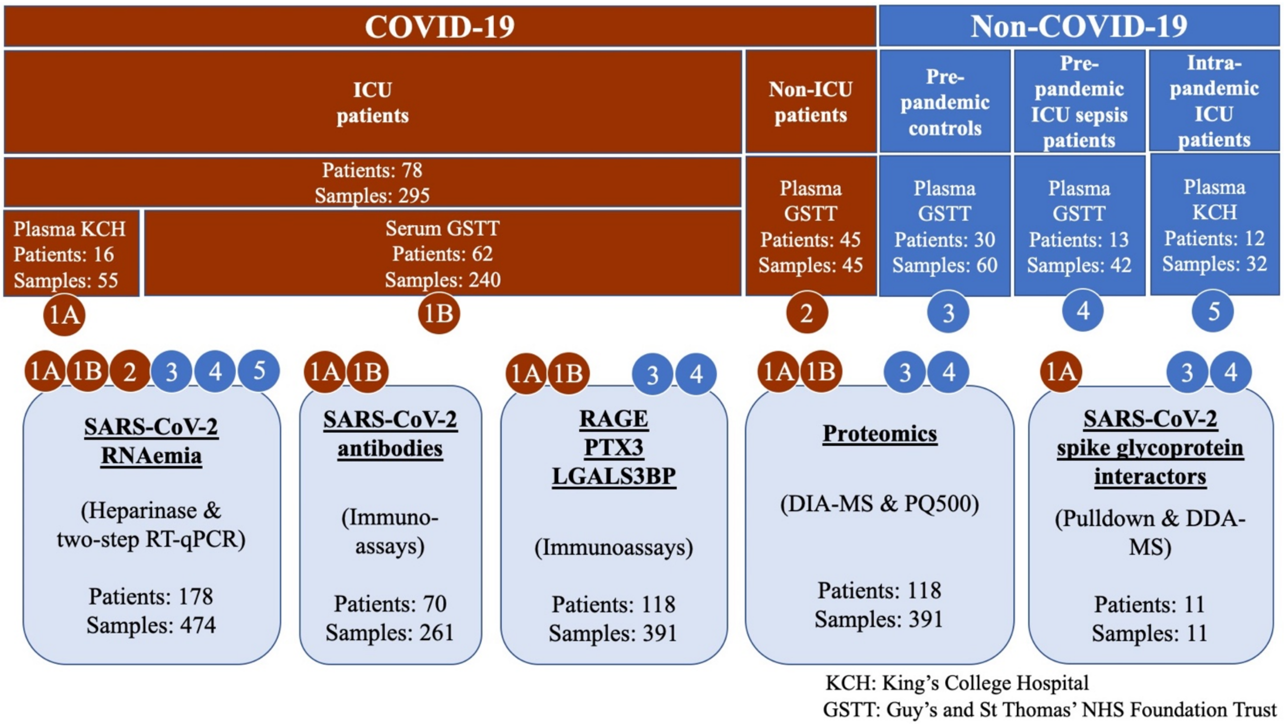

Supplementary Fig. 1: Study design.

a

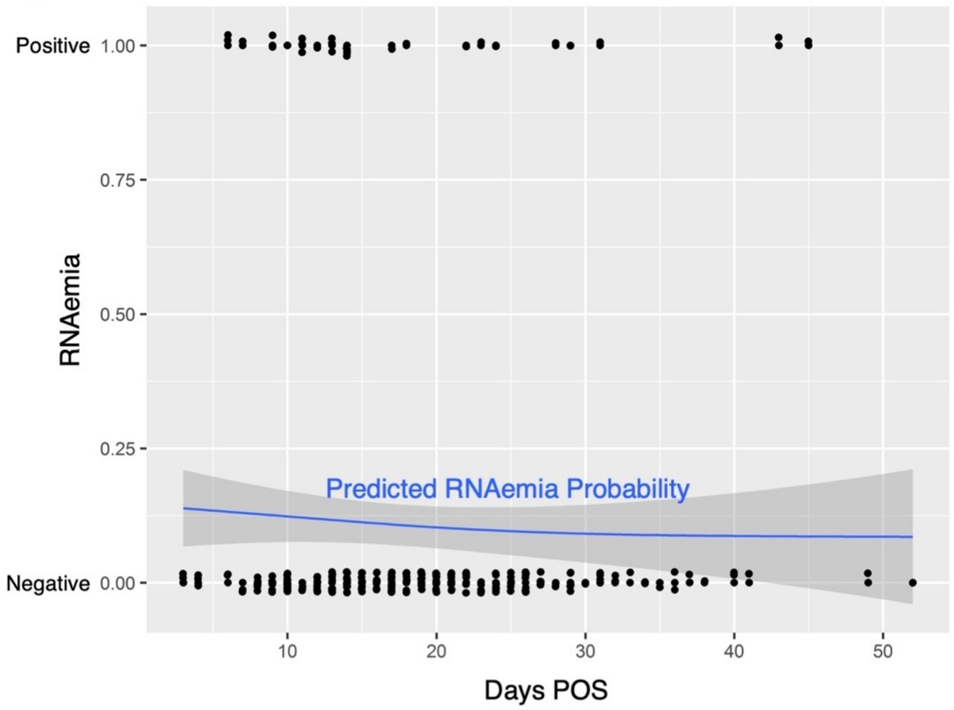

b

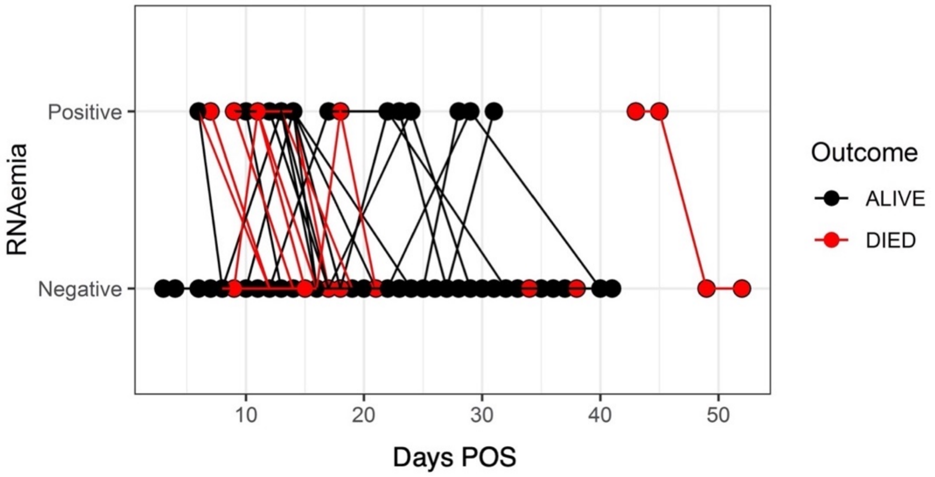

***Supplementary Fig. 2: RNAemia frequency and time from symptom onset in COVID-19 ICU patients (n = 78). a**, Shown are the 95% CI of the predicted RNAemia probability (grey band) with days post onset of symptoms (POS) alongside individual RNAemia positive and negative results. **b**, RNAemia trajectory of individual patients is shown over time (days POS). Red - died, black – alive.*

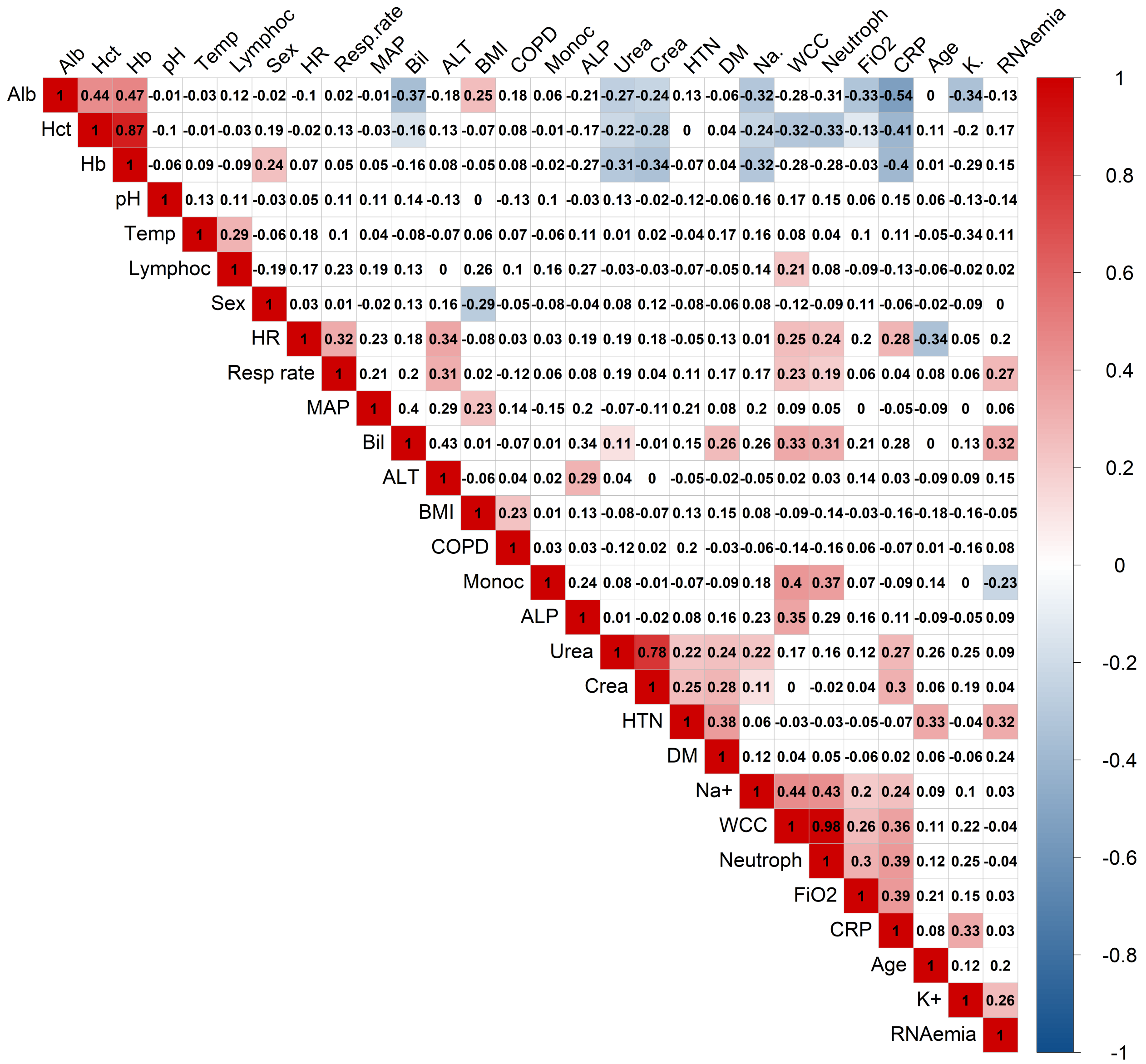

Supplementary Fig. 3: Pairwise Spearman correlation for continuous variables, Cohen's Kappa for pairwise correlation of binary variables and point-biserial correlation for pairwise correlation of continuous and binary variables in 78 COVID-19 ICU patients at baseline. *Hierarchical clustering analysis and heat-map matrix illustrates positive and negative co-expression and clusters. Three distinct clusters emerge: one comprising of albumin, hematocrit and hemoglobin, a second cluster with urea, creatinine, hypertension, and type 2 diabetes and a third cluster with sodium, WCC, neutrophils, FiO_2 and CRP. White indicates no significant correlation (P value >0.05). Red indicates positive and blue negative correlation with P value <0.05. Abbreviations: Alb: albumin, ALP: alkaline phosphatase, ALT: alanine aminotransferase, Bil: bilirubin, Crea: creatinine, CRP: C-reactive protein, DM: type 2 diabetes, Hct: hematocrit, Hb: hemoglobin, HR: heart rate, HTN: hypertension, Lymphoc: lymphocytes, MAP: mean arterial pressure, Monoc: monocytes, Neutroph: neutrophils, K: potassium, Resp. rate: respiratory rate, Na.: sodium, Temp: body temperature, WCC: white cell count. All statistical analyses are two-tailed.*

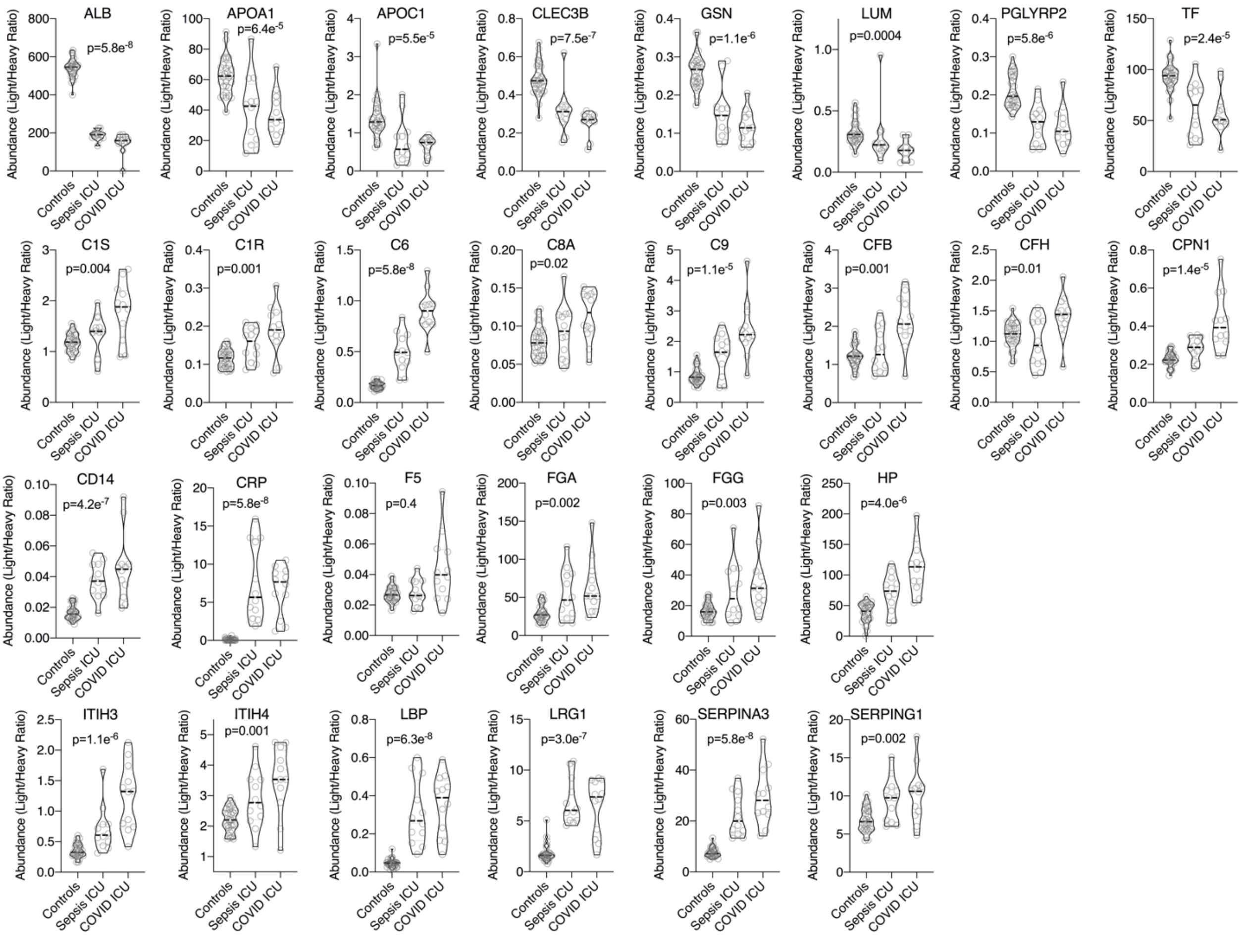

Supplementary Fig. 4: COVID-19 plasma proteome signature, common to published reports. *Plasma proteome profiling was conducted using a DIA-MS approach with spiked authentic heavy standards. 100 proteins were significantly different in the plasma proteome of COVID-19 ICU patients (n = 12), when compared to control (n = 30) and sepsis ICU patients (n = 12), and this panel was cross-referenced against two previously published proteomic studies, exploring circulating protein markers of COVID-19 severity, to generate a panel of 29 common proteins. These 29 common proteins, except LGALS3BP, are each individually represented to highlight their variation across disease phenotype. Significance was determined through the Kruskal-Wallis test with Benjamini and Hochberg's FDR correction.*

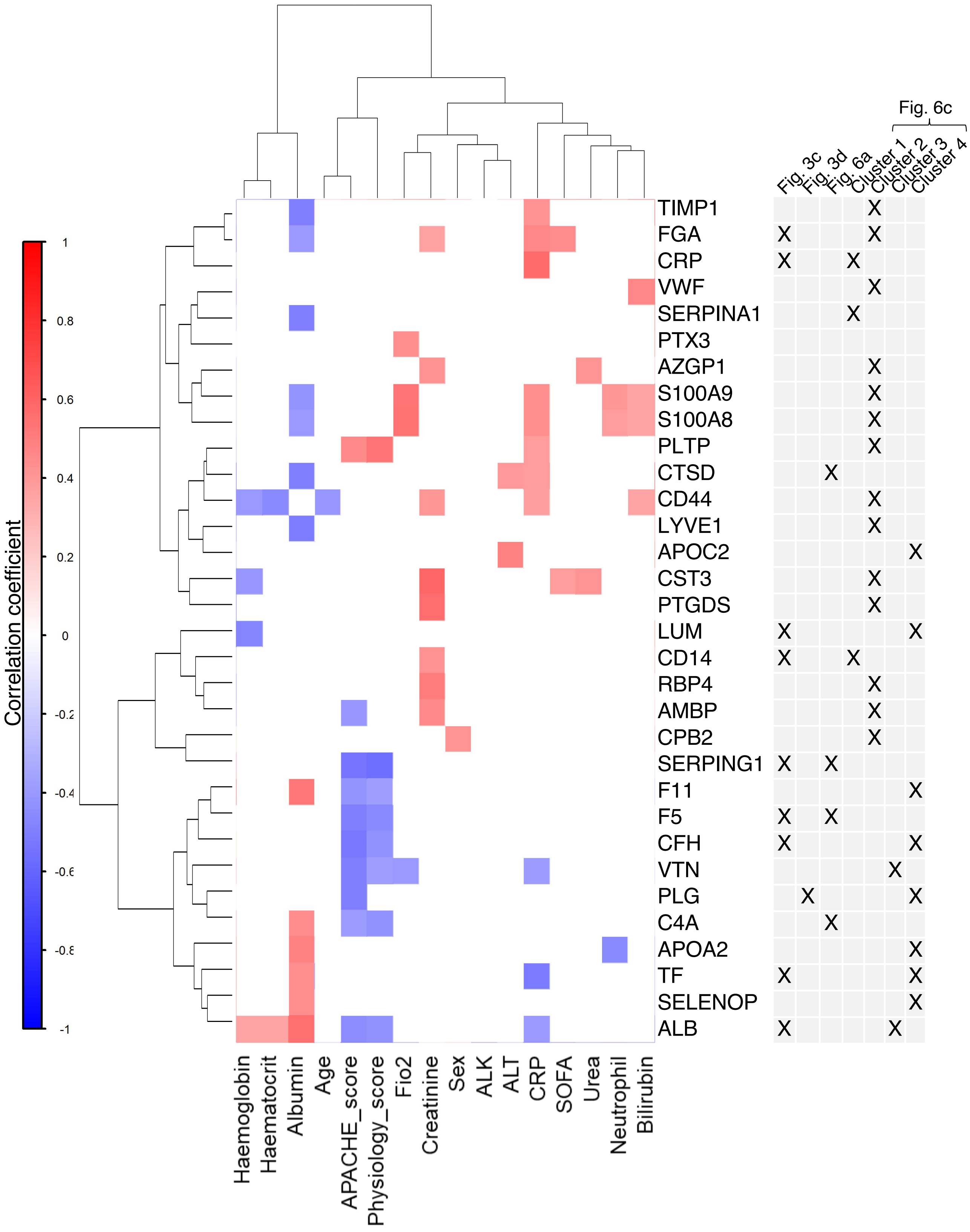

Supplementary Fig. 5: Correlation of baseline clinical variables with serum proteins in COVID-19 ICU patients (n = 62). *Only proteins are shown that had at least one significant correlation (q-value < 0.05) with a clinical variable and were presented in Fig. 3c, Fig. 3d, Fig. 6a, Fig. 6c. PTX3 is also shown. PTX3 levels are based on ELISA measurements, all other proteins are based on DIA-MS analysis. Spearman correlation and point biserial correlation were used to calculate correlations against continuous and binary variables,*

respectively. The Benjamini-Hochberg FDR adjustment was applied row-wise to correct for multiple testing. Hierarchical bi-clustering was applied to rank the proteins and clinical variables. Outliers were visually inspected in the correlograms and removed for each correlation after performing the Grubbs test to confirm the presence of an outlier. Statistical analysis is two-tailed.

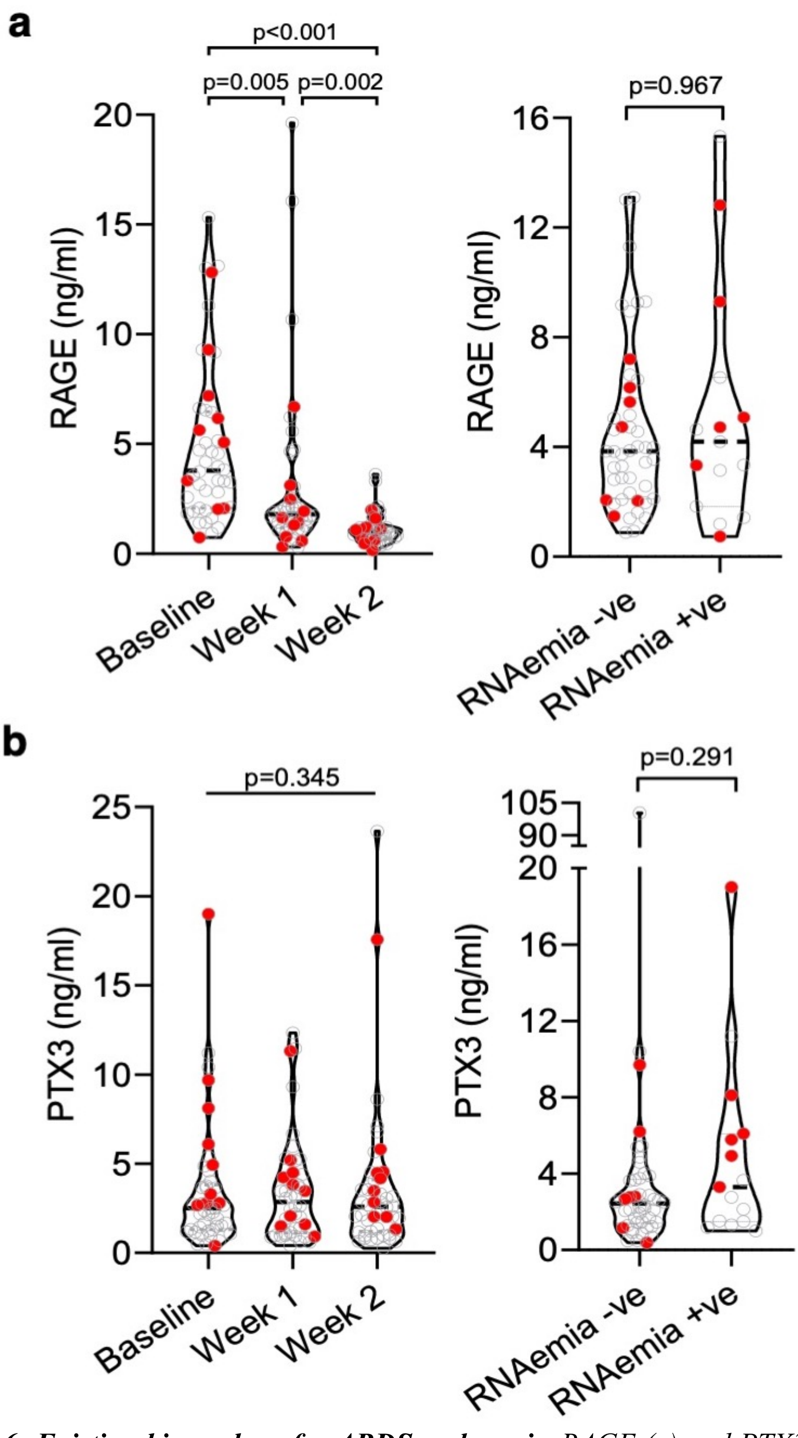

Supplementary Fig. 6: Existing biomarkers for ARDS and sepsis. *RAGE (**a**) and PTX3 (**b**) were measured by ELISA. Longitudinal comparisons at baseline, week 1 and week 2 after admission to the GSTT ICU (n = 46). Red dots highlight patients who died, white dots represent patients who survived. Friedman test with Dunn's multiple comparisons was used to determine statistical significance. Comparisons according to RNAemia status (n = 46 negative: "-ve"; n = 15 positive: "+ve"). Significance was determined by a two-tailed Mann-Whitney U test, correcting for age and sex.*

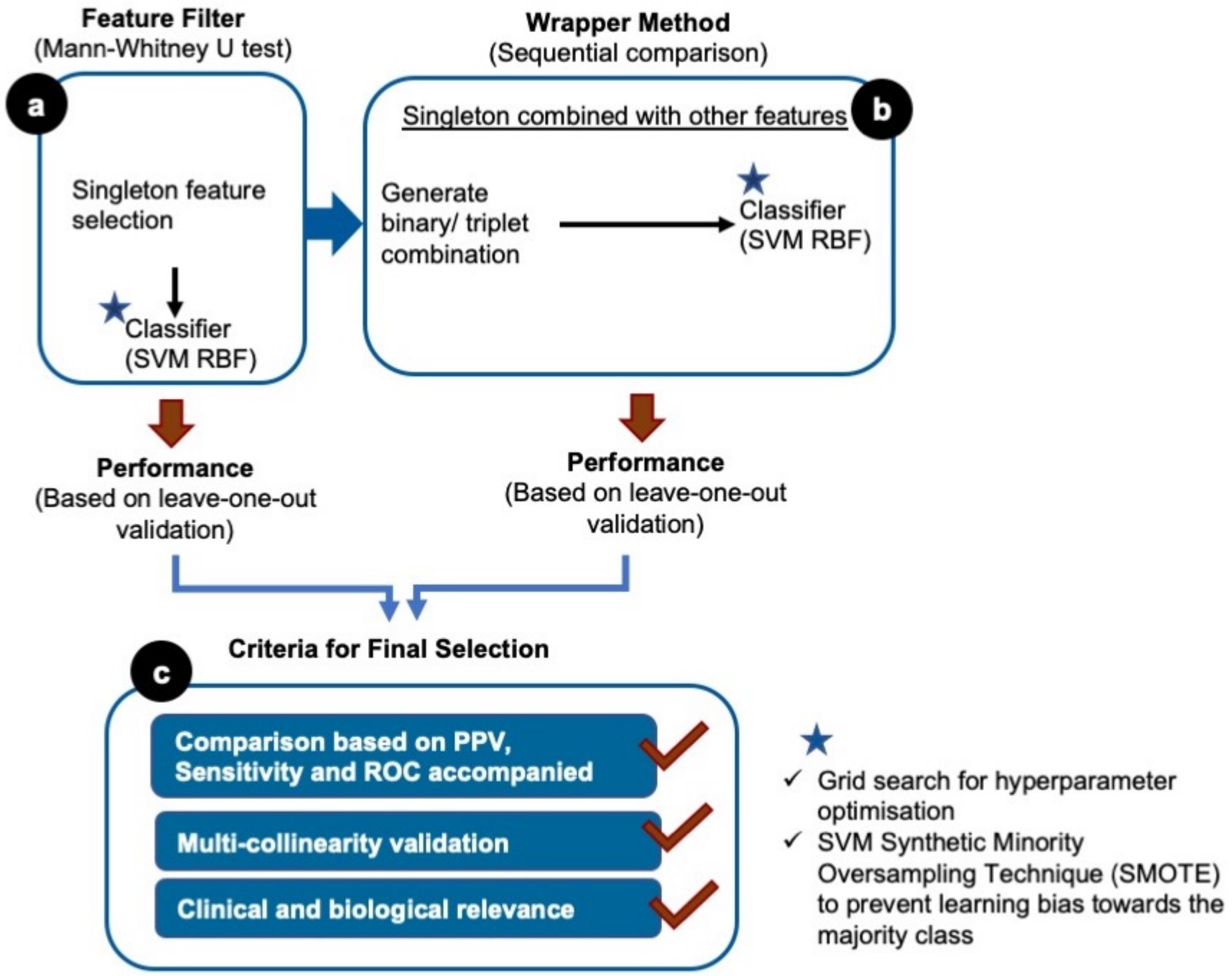

***Supplementary Fig. 7: Illustration of feature and model selection process for the support vector machine (SVM) approach using both GSTT and KCH cohorts (n = 78). a**, As a first step, singleton features were selected based on statistical significance with P value <0.05. The performance of these singleton markers was measured using SVM with RBF (radial basis function) kernel. **b**, As a second step, the combinations for the shortlisted singleton features were restricted to a maximum of triplets to enhance ease of clinical implementation and avoid the risk of overfitting. **c**, Final signature(s) selection from singleton (step a) and combinations (step b) was undertaken based on classifier performance, multi-collinearity validation and clinical relevance. This study data is imbalanced reflecting the true population scenario in COVID-19 ICU patients. Hence, for classifier performance, average of sensitivity, PPV and ROC AUC was used. While the F1-score, i.e. the harmonic mean of sensitivity and precision (PPV) is a commonly used evaluation metric for imbalanced data, the drawback of the F1-score is that it does not reflect the correct classification of the majority class, i.e. true negatives. Hence, PPV and sensitivity in combination with ROC AUC were used to addresses this limitation of standalone usage of F1-score. Multi-collinearity validation was conducted using pairwise correlation - Spearman for continuous variables, point bi-serial for pairwise continuous and binary variables and Kappa correlation for pairwise binary variables.*

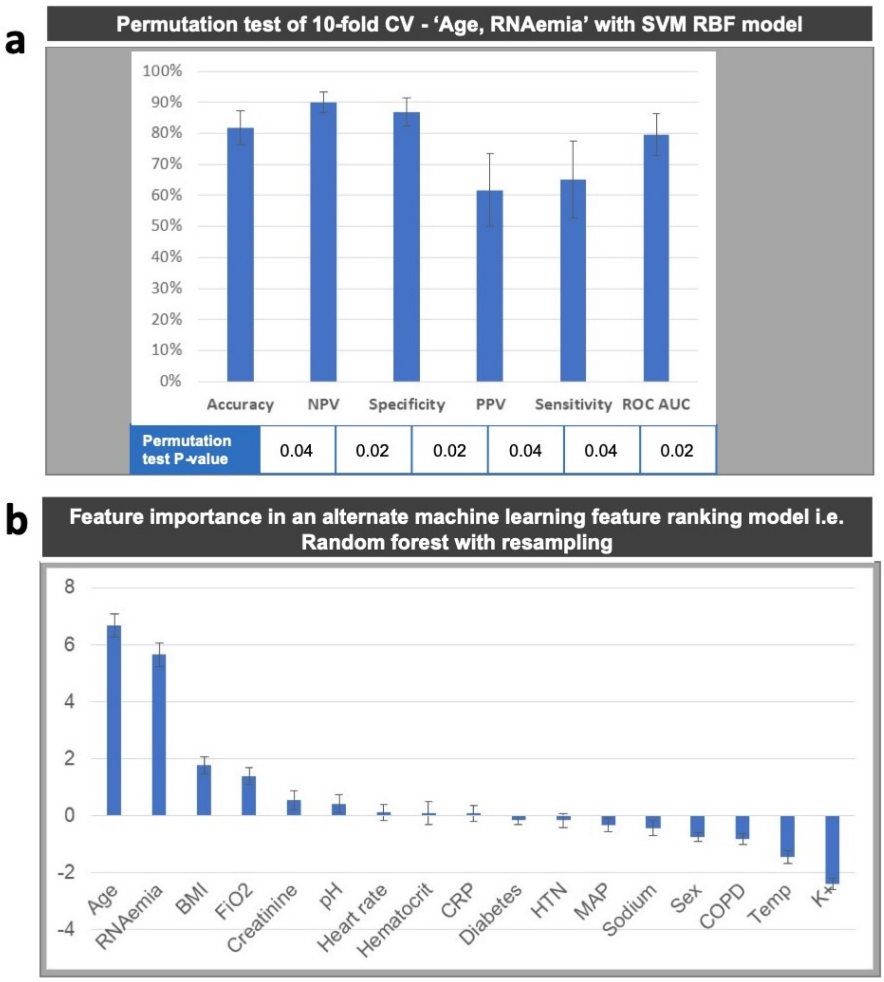

Supplementary Fig. 8: Technical validation of machine learning model. a, *Permutation test of SVM classifier performance: 10-fold cross-validation (CV) of the best predictor showing mean performance and standard error of the mean. 10-fold CV is comparable with leave-one-out metrics shown in Supplementary table 8. Significance test of 10-fold CV performed using permutation test with 50 permutes, i.e. repeating the classification procedure after random permuting of the outcome labels. This returned a significant P value, (i.e. two-tailed permutation test P value <0.05), thus indicating that the classifier has found a real class structure (pattern) in the data. Hence, rejecting the null hypothesis that the classifier performance is by chance, i.e. input variables and outcome labels are independent.* ***b,*** *Stability of feature importance under a Random Forest (RF) Model showing mean variable importance and standard error of mean across 100 resampling cycles of sensitivity analysis. Each resampling cycle takes equal proportions of the two outcome classes from the complete training data. Top 17 (of the 28) features are shown based on mean importance. The features with negative scores make the prediction worse and should be excluded from the model. The technical validation of feature importance stability using this alternate machine learning method, i.e. RF with resampling reinforces the importance of best predictor, i.e. 'Age, RNAemia' that are ranked among the top 2 most important features. Abbreviations: CRP: C-reactive protein, HTN: Hypertension, MAP: Mean arterial pressure,* K^+*: potassium, Temp: Body temperature.*

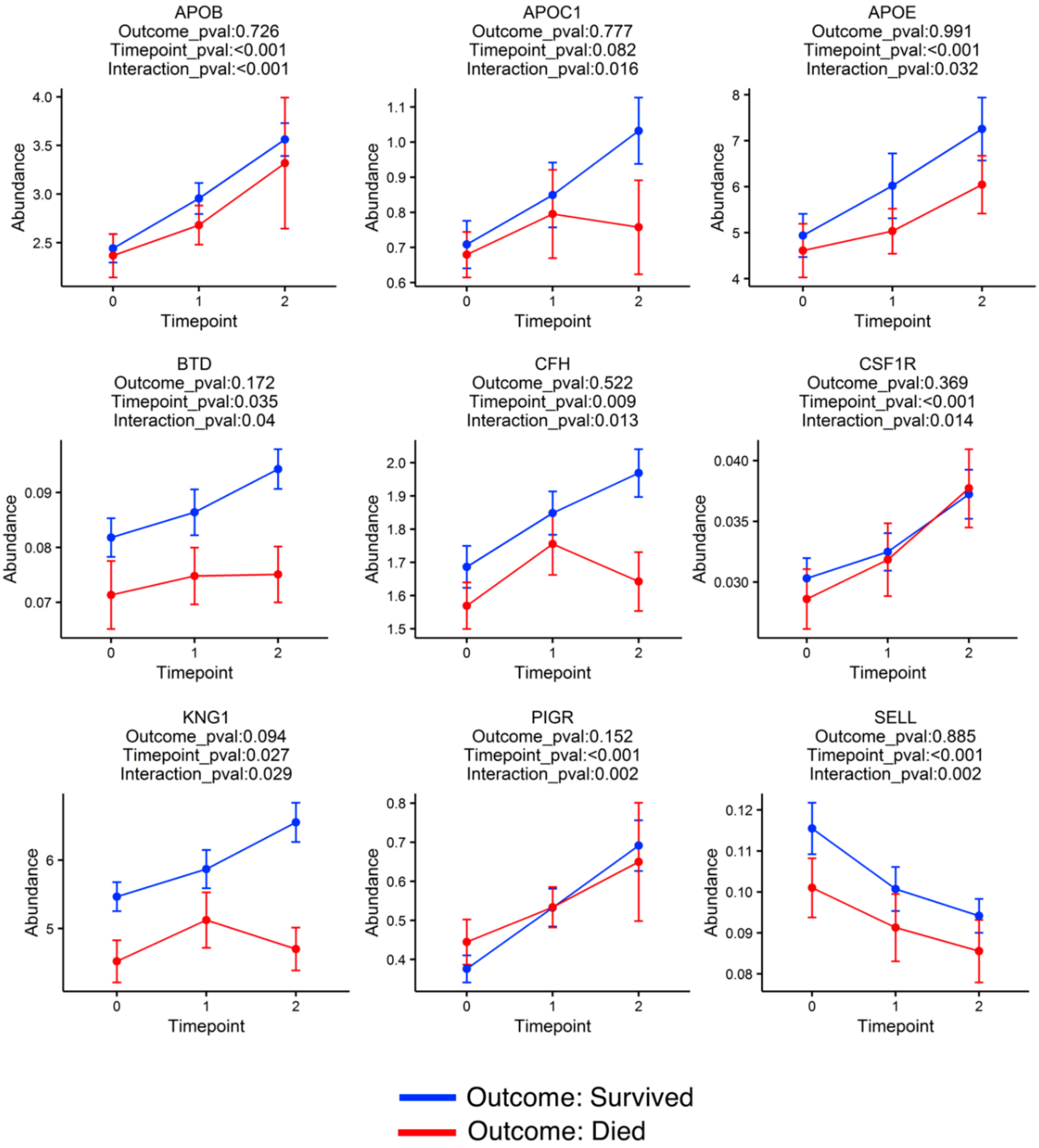

Supplementary Fig. 9: Comparison of the trajectories of individual proteins in COVID-19 ICU patients (non-survivors: n = 10, survivors: n = 37). *Listed are the proteins that show a significant interaction between survival and time of measurement (0 = ICU admission; 1 = first week after ICU admission; 2 = second week after ICU admission). Linear Mixed Models analysis was performed to calculate the P values, correcting for age and sex. Abbreviations: ApoB, apolipoprotein B; ApoC1, apolipoprotein C1; ApoE, apolipoprotein E; BTD, biotinidase; CFH, complement factor H; CSF1R, macrophage colony-stimulating factor 1 receptor; KNG1, kininogen; SELL, L-selectin. Median and 95% CI of median are shown.*

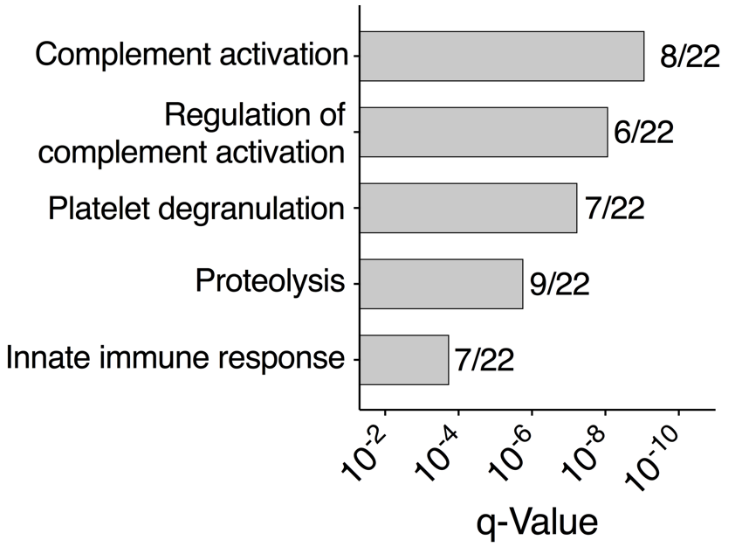

Supplementary Fig. 10: LGALS3BP enriched functional pathways. *Proteins that correlated with LGALS3BP levels in COVID-19 ICU patients (n = 12) as determined by proteomics with a greater than 0.6 Spearman correlation coefficient were analyzed to determine enriched functional pathways. Gene ontology analysis revealed an enrichment of protein pathways related to the complement system, platelet degranulation, proteolysis and the innate immune response.*

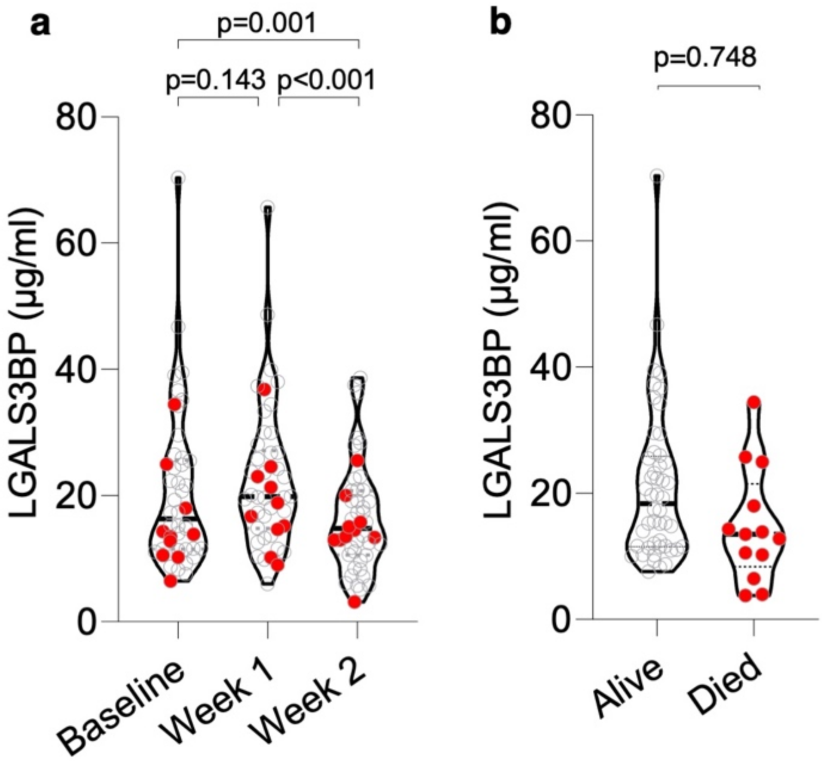

Supplementary Fig. 11: LGALS3BP in COVID-19 patients as determined by ELISA. a*, Longitudinal comparisons at baseline, week 1 and week 2 after admission to the GSTT ICU (n = 46). Red dots highlight patients who died, white dots represent patients who survived. Friedman test with Dunn's multiple comparisons test was used to determine statistical significance, correcting for age and sex.* ***b****, Comparisons based on 28-day ICU mortality (n = 48 alive; n = 13 died). Significance was determined by a two-tailed Mann-Whitney U test, correcting for age and sex.*

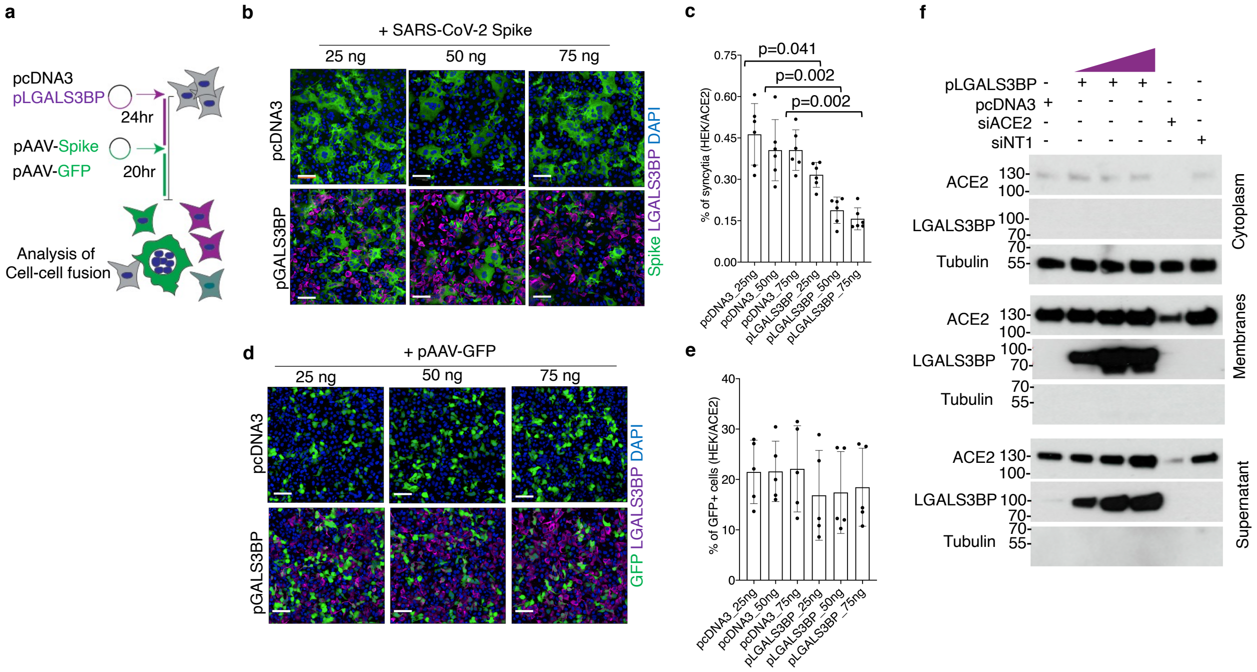

Supplementary Fig. 12: LGALS3BP overexpression impairs spike-mediated syncytia formation in a dose-dependent manner. ***a****, Schematic representation of the SARS-CoV-2 spike-mediated cell-cell fusion assay.* ***b, c,*** *HEK293-ACE2 cells were transfected with different concentrations of either pcDNA3 or pLGALS3BP, followed by transfection of pAAV-Spike 24 hours later. After 20 hours, cells were immunostained with anti-LGALS3BP (violet), anti-Spike (green) and DAPI for nuclei (blue). Representative images are shown in (****b****), and quantifications in (****c****), where data are plotted as the percentage of fused cells, normalized to the total number of cells (mean ± standard deviation; n = 6; Mann-Whitney U test). Scale bars in (****b****) represent 100 µm.* ***d, e****, HEK293-ACE2 cells were transfected with different concentrations of either pcDNA3 or LGALS3BP, followed by transfection of the GFP expressing vector 24 hours later. After 20 hours, cells were immunostained with anti-LGALS3BP (violet), anti-GFP (green) and DAPI for nuclei (blue). Representative images are shown in (****d****), and quantifications are shown in (****e****), where data are plotted as the percentage of GFP-positive cells, normalized to the total number of cells (mean ± standard deviation; n = 4). Scale bars in (****d****) represent 100 µm.* ***f****, Expression levels of LGALS3BP and ACE2. HEK293-ACE2 cells were transfected with different concentrations of pLGALS3BP, pcDNA3 and ACE2 siRNAs. After 48 hours, supernatants were collected, and cells were used for cellular fractionation. Levels of LGALS3BP, ACE2 and tubulin were assessed by immunoblotting with anti-LGALS3BP, anti-tubulin and anti-ACE2 antibodies, respectively. Lack of tubulin immunoreactivity in the indicated supernatants and membrane fractions indicates the absence of detectable cell lysis and purity of cellular fractionation. Molecular weight markers (kDA) are displayed on the left side of the immunoblots. Immunoblots were performed in two independent experiments. All statistical analyses are two-tailed.*

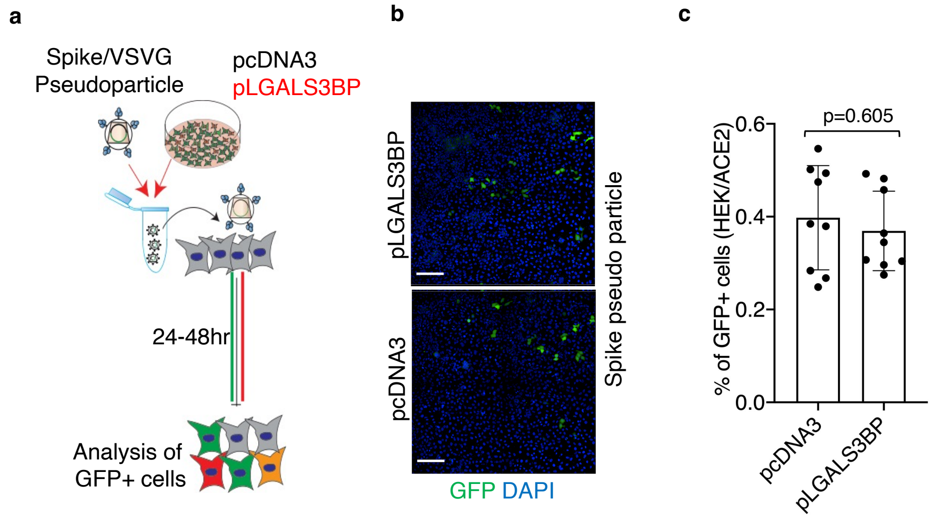

***Supplementary Fig. 13: Supernatant from LGALS3BP-expressing cells does not impair SARS-CoV-2 spike-pseudoparticle entry. a**, Schematic representation of the procedure to assess the activity of supernatant from LGALS3BP expressing cells on spike pseudoparticles. Spike pseudoparticles carrying GFP as a reporter were mixed with cell culture supernatant from pLGALS3BP and pcDNA3 transfected cells for 15 minutes before transducing HEK293-ACE2 cells. After 36 hours, cells were immunostained with anti-GFP (green) and DAPI for nuclei (blue). Representative images are shown in (**b**) and quantifications are shown in (**c**). Data (mean ± standard deviation; n = 9; two-tailed Mann-Whitney U test) are plotted as the percentage of GFP-positive cells normalized to the total number of cells. Scale bars represent 200 µm.*

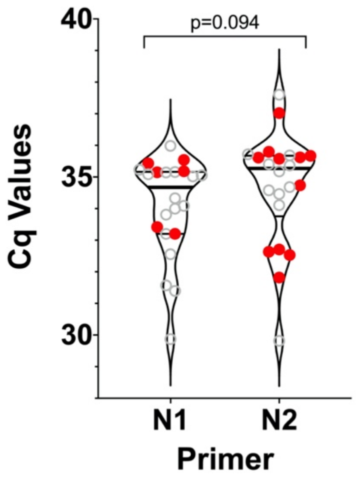

***Supplementary Fig. 14: Abundance of SARS-CoV-2 RNA in plasma or serum**. Cq values of all samples in which SARS-CoV-2 RNA was detectable (Cq <40) are shown. Comparison between N1 and N2 Cq values is based on a two-tailed Mann-Whitney U test. Red - died, white - survived.*

Supplementary Table 1: Baseline characteristics of COVID-19 ICU patients.

COVID-19 Clinical Characteristics	COVID-19 ICU Patients (n = 78)	COVID-19 'Survivors' (n = 60)	COVID-19 'Non-Survivors' (n = 18)	P value
SARS-COV-2 RNAemia (%)	23.08%	13.33%	55.56%	<0.001
Days POS until ICU Admission	7.00 (6.25, 10.00)	7.00 (6.00, 10.00)	9.50 (7.00, 14.00)	0.099
Days POS until Death	-	-	22.00 (19.00, 34.00)	-
Days from Admission to Death	-	-	13.50 (11.00, 15.50)	-
**Demographics**				
Age (Years)	54.00 (46.25, 64.01)	52.01 (44.00, 61.01)	65.01 (57.51,77.04)	<0.001
Sex (% Male)	71.79%	70.00%	77.78%	0.766
BMI (kg/m^2)	28.05 (24.71, 34.28)	28.10 (24.80, 34.90)	25.87 (24.55, 31.46)	0.252
**Comorbidities**				
COPD (%)	16.67%	15.00%	22.22%	0.483
Diabetes (%)	25.64%	25.00%	27.78%	0.769
Hypertension (%)	37.18%	33.33%	50.00%	0.267
**Acute Care Parameters**				
APACHE II Score	15.00 (11.00,19.00)	14.00 (11.00, 18.00)	17.00 (13.75, 20.50)	0.126
SOFA Score	6.00 (4.00, 8.00)	6.00 (4.00, 7.00)	8.00 (4.00, 9.00)	0.171
FiO_2 (Fraction of 1)	0.50 (0.35, 0.60)	0.47 (0.35, 0.56)	0.52 (0.50, 0.60)	0.113
Heart rate (bpm)	96.01 (63.27, 112.49)	96.50 (63.01, 114.99)	93.00 (86.00, 101.51)	0.767
MAP (mmHg)	65.51 (61.09, 79.75)	65.17 (61.09, 80.75)	65.51 (61.33, 70.50)	0.749
Respiratory rate (bpm)	22.00 (16.25, 28.07)	20.7 (16.00, 28.00)	23.83 (22.00, 28.77)	0.138
Temperature (°C)	38.19 (36.85, 39.07)	38.44 (36.95, 39.02)	37.94 (36.85, 38.97)	0.656
**Blood Biochemistry**				
Albumin (g/L)	30.00 (27.01, 33.00)	30.00 (27.01, 33.00)	29.50 (26.26, 30.28)	0.167
ALP (U/L)	64.49 (48.99, 94.49)	64.49(48.74, 97.24)	61.00 (50.25, 80.75)	0.981
ALT (IU/L)	36.01 (27.02, 51.92)	35.49 (25.02, 47.31)	41.68 (30.47, 55.86)	0.171
Bilirubin (µmol/L)	11.49 (8.00, 25.91)	10.99 (8.00, 21.89)	18.02 (8.00, 34.75)	0.313
Creatinine (µmol/L)	94.49 (72.02, 170.03)	89.38 (69.77, 174.54)	124.03 (91.24, 153.75)	0.265
C-reactive protein (mg/L)	226.21 (143.02, 328.21)	237.71 (103.56, 336.98)	215.68 (167.97, 299.57)	0.981
Hemoglobin (g/L)	115.63 (103.26, 123.75)	114.50 (100.51, 121.25)	120.00 (107.76, 125.74)	0.224
pH	7.36 (7.31, 7.41)	7.36 (7.31, 7.42)	7.32(7.31, 7.38)	0.233
Potassium (mmol/L)	4.50 (4.30, 4.80)	4.50 (4.30, 4.80)	4.35 (4.12, 4.85)	0.668
Sodium (mmol/L)	140.00 (137.00, 143.99)	140.00 (137.00, 143.24)	140.50 (137.25, 144.99)	0.426
Urea (mmol/L)	7.11 (5.01, 12.37)	6.55 (4.87, 10.77)	10.70 (6.36, 15.27)	0.039
**Blood Count**				
Hematocrit (%)	37.20 (30.26, 39.82)	36.00 (29.71, 39.30)	39.30 (35.25, 41.10)	0.069
Lymphocytes (10^9/l)	0.86 (0.63, 1.20)	0.88 (0.69, 1.20)	0.83 (0.45, 1.09)	0.498
Monocytes (10^9/l)	0.40 (0.25, 0.50)	0.36 (0.28, 0.51)	0.40 (0.24, 0.48)	0.743
Neutrophils (10^9/l)	7.38 (4.90, 9.68)	7.27 (4.90, 9.53)	7.50 (4.29, 9.83)	0.934
White cell count (10^9/l)	8.50 (6.53, 11.28)	8.50 (6.60, 11.00)	8.53 (5.95, 12.12)	0.962

Continuous variables are presented as median (25th and 75th percentile). P value computed for survivors vs non-survivors using Mann-Whitney test for continuous variables and Fisher exact test for binary variables (both two-tailed). SARS-CoV-2 RNAemia: positive blood test within first six days of admission to ICU. Baseline characteristics shown in patients from GSTT and KCH cohorts. Abbreviations: Days POS: days post onset of symptoms; APACHE II score: acute physiology and chronic health evaluation II score; SOFA score: the sequential organ failure assessment score; MAP: mean arterial pressure; ALP: alkaline phosphatase, ALT: alanine aminotransferase.

Supplementary Table 2: Baseline characteristics of non-ICU, hospitalized COVID-19 patients.

Clinical Characteristics	Non-ICU COVID-19 patients (n = 45)
SARS-CoV-2 RNAemia (%)	4.44%
Non-Survivors 28 days after hospitalization (%)	11.11%
**Demographics**	
Age (years)	60.82 (45.95, 71.58)
Sex (% Male)	66.66%
BMI (kg/m^2)	26.32 (21.32, 31.50)
**Comorbidities**	
COPD – no. (%)	8 (17.77%)
Diabetes – no. (%)	12 (26.66%)
Hypertension – no. (%)	21 (46.66%)
**Acute Care Parameters**	
FiO_2 (Fraction of 1)	0.21 (0.21, 0.28)
Heart rate (bpm)	103 (87, 118)
Temperature (°C)	37.6 (36.85, 38.3)
**Blood Biochemistry**	
Albumin (g/L)	37.00 (32.75, 40.00)
ALT (IU/L)	30.50 (16.50, 52.00)
Bilirubin (µmol/L)	8 (4.5, 11)
Creatinine (µmol/L)	71 (62, 89)
C-reactive protein (mg/L)	58 (29, 138)
**Blood Count**	
Lymphocytes (10^9/l)	1.05 (0.63, 1.30)
Monocytes (10^9/l)	0.50 (0.40, 0.70)
Neutrophils (10^9/l)	5.15 (3.33, 7.00)
Platelets (10^9/l)	261.5 (192.0, 334.5)
White cell count (10^9/l)	6.90 (4.65, 9.28)

Continuous variables are presented as median (25th and 75th percentile). Abbreviations: ALT: alanine aminotransferase.

Supplementary Table 3: Baseline characteristics of COVID-19 ICU patients with and without RNAemia.

COVID-19 Clinical Characteristics	COVID-19 ICU Patients (*n* = 78)	RNAemia Negative (*n* = 60)	RNAemia Positive (*n* = 18)	*P* value
Non-Survivors (%)	23.08%	13.33%	55.56%	<0.001
Days POS until ICU Admission	7.00 (6.25, 10.00)	7.00 (6.00, 10.00)	7.00 (6.25, 10.00)	0.690
Days POS until Death	22.00 (19.00, 34.00)	26.00 (19.00, 36.50)	21.50 (18.50, 25.25)	0.434
Days from Admission to Death	13.50 (11.00, 15.50)	13.00 (12.00, 17.00)	14.00 (11.00, 15.00)	0.641
**Demographics**				
Age (Years)	54.00 (46.25, 64.01)	53.01 (45.75, 62.26)	61.01 (48.25, 66.01)	0.107
Sex (% Male)	71.79%	71.67%	72.22%	1.000
BMI (kg/m^2)	28.05 (24.71, 34.28)	28.05 (24.65, 34.68)	29.38 (24.93, 31.70)	0.845
**Comorbidities**				
COPD (%)	16.67%	15.00%	22.22%	0.483
Diabetes (%)	25.64%	20.00%	44.44%	0.062
Hypertension (%)	37.18%	28.33%	66.67%	0.005
**Acute Care Parameters**				
APACHE II Score	15.00 (11.00, 19.00)	14.50 (11.00, 17.75)	17.00 (11.75, 23.00)	0.114
SOFA Score	6.00 (4.00, 8.00)	5.00 (4.00, 8.00)	7.00 (4.00, 9.00)	0.363
FiO_2 (Fraction of 1)	0.50 (0.35, 0.60)	0.50 (0.35, 0.60)	0.50 (0.46, 0.59)	0.306
Heart rate (bpm)	96.01 (63.27, 112.49)	93.50 (62.76, 114.25)	99.50 (92.50, 108.25)	0.140
MAP (mmHg)	65.51 (61.09, 79.75)	63.50 (59.08, 80.08)	67.67 (65.34, 70.76)	0.057
Respiratory rate	22.00 (16.25, 28.07)	20.75 (13.80, 28.00)	26.00 (22.00, 30.00)	0.023
Temperature (°C)	38.19 (36.85, 39.07)	38.29 (36.05, 39.09)	38.19 (37.75, 38.99)	0.622
**Blood Biochemistry**				
Albumin (g/L)	30.00 (27.01, 33.00)	30.50 (27.01, 33.00)	29.50 (27.01, 30.00)	0.178
ALP (U/L)	64.49 (48.99, 94.49)	63.49 (48.99, 94.98)	65.51 (49.24, 88.49)	0.896
ALT (IU/L)	36.01 (27.02, 51.92)	33.00 (25.02, 43.31)	51.62 (41.02, 60.75)	0.003
Bilirubin (µmol/L)	11.49 (8.00, 25.91)	10.99 (8.00, 17.27)	30.47 (10.14, 44.17)	0.011
Creatinine (µmol/L)	94.49 (72.02, 170.03)	93.49 (71.53, 166.00)	100.51 (81.03, 168.03)	0.817
C-reactive protein (mg/L)	226.21 (143.02, 328.21)	237.71 (138.78, 336.88)	189.52 (153.54, 320.12)	0.910
Hemoglobin (g/L)	115.63 (103.26, 123.75)	113.85 (100.51, 121.25)	119.50 (114.00, 126.00)	0.104
pH	7.36 (7.31, 7.41)	7.36 (7.31, 7.42)	7.32 (7.30, 7.38)	0.217
Potassium (mmol/L)	4.50 (4.30, 4.80)	4.40 (4.30, 4.72)	4.65 (4.27, 4.98)	0.118
Sodium (mmol/L)	140.00 (137.00, 143.99)	140.00 (137.00, 143.99)	139.50 (137.25, 144.49)	0.794
Urea (mmol/L)	7.11 (5.01, 12.37)	6.55 (4.80, 11.77)	8.42 (6.63, 14.55)	0.141
**Blood Count**				
Hematocrit (%)	37.20 (30.26, 39.82)	36.00 (29.63, 39.30)	38.55 (35.85, 40.57)	0.078
Lymphocytes (10^9/l)	0.86 (0.63, 1.20)	0.80 (0.64, 1.19)	0.88 (0.62, 1.23)	0.784
Monocytes (10^9/l)	0.40 (0.25, 0.50)	0.40 (0.30, 0.59)	0.30 (0.21, 0.40)	0.064
Neutrophils (10^9/l)	7.38 (4.90, 9.68)	7.34 (4.90, 9.53)	7.69 (4.16, 10.27)	0.896
White cell count (10^9/l)	8.50 (6.53, 11.28)	8.50 (6.58, 11.00)	9.40 (5.36, 12.16)	0.995

Continuous variables are presented as median (25th and 75th percentile). P value computed for SARS-CoV-2 RNAemia positive vs negative patients within first six days of admission to ICU using Mann-Whitney U test for continuous variables and Fisher exact test for binary variables (both two-tailed). Baseline characteristics shown in patients from GSTT and KCH cohorts. Abbreviations: Days POS: days post onset of symptoms; APACHE II score: acute physiology and chronic health evaluation score; SOFA score: the sequential organ failure assessment score; MAP: mean arterial pressure; ALP: alkaline phosphatase, ALT: alanine aminotransferase.

Supplementary Table 4: Baseline characteristics of non-COVID-19 patients.

Clinical Characteristics	(A) Non-COVID-19, non-ICU controls (*n* = 30)	(B) Pre-pandemic, non-COVID-19 ICU sepsis patients (*n* = 13)	(C) Intra-pandemic, non-COVID-19 ICU patients (*n* = 12)	(A) *vs* (B) *P* value	(A) *vs* (C) *P* value	(B) *vs* (C) *P* value
SARS-CoV-2 RNAemia (%)	0%	0%	0%	1.0	1.0	1.0
Non-Survivors at day 28 (%)	-	0%	5 (41.66%)	-	-	0.0149
**Demographics**						
Age (Years)	70 (64.00, 74.75)	64 (38, 73)	70 (59.25, 78)	0.1857	0.8563	0.3694
Sex – % Male	22 (73.33%)	7 (53.85%)	7 (58.33%)	0.2917	0.4635	1.0
BMI (kg/m^2)	28.22 (25.02, 30.77)	26.12 (23.88, 27.55)	30.30 (26.77, 34.09)	0.1774	0.3749	0.08213
**Comorbidities**						
COPD (%)	6 (20%)	3 (23.08%)	3 (25%)	1.0	0.6987	1.0
Diabetes (%)	7 (23.33%)	2 (15.39%)	6 (50%)	0.6989	0.1405	0.0968
Hypertension (%)	17 (56.66%)	7 (53.85%)	5 (41.66%)	1.0	0.4994	0.6951
**Acute Care Parameters**						
APACHE II score	-	16 (15, 21)	-	-	-	-
FiO_2 (Fraction of 1)	-	0.4 (0.27, 0.65)	0.28 (0.25, 0.31)	-	-	0.107
Heart rate (bpm)	-	115 (69, 136)	80 (78, 93)	-	-	0.1652
MAP (mmHg)	-	65.67 (60, 99.33)	81.5 (72, 85.75)	-	-	0.1653
Respiratory rate	-	23.50 (19.00, 28.75)	20.5 (19.75, 25.25)	-	-	0.4688
SOFA score	-	-	3.5 (1.75, 6.25)	-	-	
Temperature (°C)	-	37.7 (35.8, 38.5)	36.9 (36.8, 37.0)	-	-	0.3404

Continuous variables are presented as median (25th and 75th percentile). P value computed using Mann-Whitney test for continuous variables and Fisher exact test for binary variables (both two-tailed). Abbreviations: APACHE II score: acute physiology and chronic health evaluation II score, MAP: mean arterial pressure. SOFA score: sequential organ failure assessment score. Intra-pandemic, non-COVID-19 ICU patients repeatedly tested negative for nasopharyngeal SARS-CoV-2 by RT-qPCR. SARS-CoV-2 RNAemia was assessed in COVID-19-negative patients to determine assay specificity.

Supplementary Table 5: External validation data based on proximity-extension assays.

Protein	Log2 Fold Change Non-Survivors vs Survivors	Average Relative Quantity	*P* value	*q* value
**PTX3**	0.89784	5.79471	<0.00001	<0.0001
**IL17C**	0.96308	2.67565	<0.00001	0.0001
**IL6**	1.42029	5.81313	<0.00001	0.0003
**PROC**	-0.37773	3.21418	<0.00001	0.0005
**IL1RN**	0.86502	2.69615	0.00001	0.001
**CX3CL1**	0.56603	4.56926	0.00003	0.002
**IL1RL1**	0.81686	2.75287	0.00004	0.003
**CCL2**	0.60728	6.13895	0.00007	0.003
**IL15**	0.47248	3.49095	0.0001	0.004
**CXCL8**	0.7077	3.27201	0.00014	0.004
**CCL20**	0.92856	5.23471	0.00032	0.007
**IL4R**	0.60584	3.31738	0.00054	0.009
**IL19**	0.67502	2.16278	0.00061	0.010
**CXCL13**	0.58454	3.52346	0.00094	0.013
**CRLF1**	0.31295	1.68468	0.00188	0.019
**F7**	-0.29250	3.56072	0.002	0.021
**CXCL10**	0.72777	7.41412	0.002	0.021
**CCL7**	0.76166	4.81714	0.003	0.025
**CCL19**	0.49678	3.5662	0.007	0.044
**IL18R1**	0.3364	6.64045	0.008	0.048
**LBP**	0.34828	4.96949	0.014	0.068
**CD14**	0.14460	7.28775	0.309	0.523
**CDH5**	0.06908	1.67967	0.343	0.554
**ITIH3**	-0.00439	2.16440	0.967	0.978

Differential expression analysis of proteins in survivors and non-survivors 28-days after hospitalization. This analysis was performed on a publicly available proximity-extension assay proteomics-based dataset (data provided by the MGH Emergency Department COVID-19 Cohort (Filbin, Goldberg, Hacohen) with Olink Proteomics: https://www.olink.com/mgh-covid-study/). Only proteins that were also quantified in our study; reported in our main findings; and cytokines and interleukins significantly associated with 28-days mortality were analyzed. Statistical analysis was conducted using the Ebayes method of the limma package, correcting for age and adjusting for multiple testing using Benjamini and Hochberg's FDR correction. Statistical analysis is two-tailed.

Supplementary Table 6: Machine learning signatures using the SVM RBF model.

Single Signature	Accuracy	NPV	Specificity	PPV (A)	Sensitivity (B)	ROC AUC (C)	Average of A+B+C
RNAemia	79.49%	86.67%	86.67%	55.56%	55.56%	85.37%	65.49%
Age	73.08%	86.79%	76.67%	44.00%	61.11%	71.11%	58.74%
PTX3	78.21%	82.09%	91.67%	54.55%	33.33%	69.35%	52.41%
Urea	65.38%	78.95%	75.00%	28.57%	33.33%	54.63%	38.84%

Binary Signature	Accuracy	NPV	Specificity	PPV (A)	Sensitivity (B)	ROC AUC (C)	Average of A+B+C
Age, PTX3	87.18%	90.32%	93.33%	75.00%	66.67%	78.52%	73.40%
Age, RNAemia	83.33%	91.23%	86.67%	61.90%	72.22%	79.81%	71.31%
Age, FiO_2	76.92%	90.38%	78.33%	50.00%	72.22%	78.70%	66.98%
Age, Albumin	82.05%	89.66%	86.67%	60.00%	66.67%	74.07%	66.91%
Age, ALT	82.05%	89.66%	86.67%	60.00%	66.67%	72.41%	66.36%
Age, Respiratory rate	80.77%	89.47%	85.00%	57.14%	66.67%	74.72%	66.18%
COPD, RNAemia	79.49%	86.67%	86.67%	55.56%	55.56%	86.39%	65.83%
Diabetes, RNAemia	79.49%	86.67%	86.67%	55.56%	55.56%	86.30%	65.80%
pH, RNAemia	73.08%	91.49%	71.67%	45.16%	77.78%	69.91%	65.30%
Hypertension, RNAemia	79.49%	86.67%	86.67%	55.56%	55.56%	82.13%	64.41%

Triplet Signature	Accuracy	NPV	Specificity	PPV (A)	Sensitivity (B)	ROC AUC (C)	Average of A+B+C
Age, FiO_2, RNAemia	84.62%	91.38%	88.33%	65.00%	72.22%	85.93%	74.38%
Age, RNAemia, PTX3	80.77%	90.91%	83.33%	56.52%	72.22%	82.59%	70.45%
Age, HR, Diabetes	87.18%	89.06%	95.00%	78.57%	61.11%	76.67%	72.12%
Age, FiO_2, PTX3	83.33%	91.23%	86.67%	61.90%	72.22%	81.85%	71.99%
Age, Sodium, PTX3	87.18%	87.88%	96.67%	83.33%	55.56%	76.20%	71.70%
Age, Lymphocytes, RNAemia	84.62%	90.00%	90.00%	66.67%	66.67%	81.20%	71.51%
Age, CRP, RNAemia	83.33%	91.23%	86.67%	61.90%	72.22%	78.98%	71.04%
Age, HR, PTX3	85.90%	87.69%	95.00%	76.92%	55.56%	80.65%	71.04%
Age, pH, RNAemia	82.05%	91.07%	85.00%	59.09%	72.22%	81.67%	70.99%
Age, Bilirubin, Hb	83.33%	91.23%	86.67%	61.90%	72.22%	78.06%	70.73%

Single markers were filtered for the prediction model based on statistical significance (P value <0.05, Supplementary Table 1, Supplementary Fig. 7). Although RNAemia surfaced as the best singleton predictor, its sensitivity was low. Age was the next best singleton predictor, however the PPV of 44% demonstrates low probability confidence in predicting mortality, i.e. the positive class. The top 10 binary and triplet combinations were selected from 114 binary and 885 triplet combinations based on the average score, i.e. average of PPV, sensitivity and ROC. While the F1-score, i.e. the harmonic mean of sensitivity and precision (PPV) is a commonly used evaluation metric for imbalanced data, the drawback is that the F1-score does not reflect the correct classification of the majority class, i.e. true negatives. Combining ROC AUC along with sensitivity and PPV addresses this limitation of standalone usage of F1-score. Signatures are shown in descending order based on average score and using leave-one-out validation. Note that PTX3 levels are different in serum and plasma. The best triplet signature 'Age, FiO_2, RNAemia' provides nominal gain in prediction probability as reflected in PPV with no uplift to specificity when compared to 'Age, RNAemia',

suggesting the binary combination to be an optimal signature to choose. Abbreviations: NPV: negative predictive value; PPV: positive predicted value; Temp: body temperature; Hb: Hemoglobin, HR: Heart rate.

Supplementary Table 7: Plasma proteins binding to SARS-CoV-2 spike glycoprotein.

Gene	Ctrl average	Spike average	Fold enrichment	*P* value	*q* value
**SPIKE**	3.35E+07	7.67E+09	228.8	0.00006	0.003
**C1QA**	1.85E+07	7.78E+07	4.2	0.007	0.037
**C1QC**	4.29E+07	1.73E+08	4	0.004	0.034
**LGALS3BP**	1.27E+06	4.59E+06	3.6	0.008	0.039
**C1QB**	2.87E+07	1.00E+08	3.4	0.022	0.057
**LSM4**	2.46E+06	8.50E+06	3.4	0.015	0.057
**C4BPB**	2.27E+07	7.05E+07	3.1	0.022	0.057
**APOD**	6.18E+06	1.58E+07	2.5	0.037	0.071
**THAP5**	1.72E+07	3.91E+07	2.2	0.028	0.062
**C4BPA**	3.89E+08	8.76E+08	2.2	0.045	0.081
**KIF20B**	7.46E+08	1.66E+09	2.2	0.011	0.047
**APCS**	1.03E+07	2.17E+07	2.1	0.022	0.057
**KIF4B**	8.84E+09	1.75E+10	1.9	0.030	0.064
**SELENOP**	1.00E+09	1.89E+09	1.8	0.020	0.057
**HRG**	4.48E+10	8.18E+10	1.8	0.038	0.071
**ZW10**	7.39E+07	1.35E+08	1.8	0.027	0.062
**HABP2**	2.02E+07	3.65E+07	1.8	0.017	0.057
**CDA**	2.12E+06	3.58E+06	1.6	0.021	0.057
**ERP44**	9.64E+05	1.48E+06	1.5	0.035	0.071
**CPN1**	3.04E+07	4.39E+07	1.4	0.024	0.058

List of the 20 non-immunoglobulin proteins detected at significantly higher levels by LC-MS/MS in pulldowns of His-tagged SARS-CoV-2 spike glycoprotein mixed with COVID-19 plasma. Control experiments were conducted to exclude: 1) co-isolates binding to the solid phase of the His-tagged pulldown metal affinity beads; 2) proteins present in the His-tagged SARS-CoV-2 preparation prior to mixing with plasma (i.e. carryover from HEK293 cells used for production of the recombinant spike glycoprotein). Significance was determined through paired t-tests with Benjamini and Hochberg's FDR correction.

Supplementary Table 8: Comparison of plasma protein binding to SARS-CoV-2 spike glycoprotein in patients with and without COVID-19.

Gene	Non-COVID-19 average	COVID-19 average	Fold enrichment	*P* value	*q* value
**LGALS3BP**	1.23E+06	4.59E+06	3.7	0.0025	0.036
**APOD**	4.95E+06	1.58E+07	3.1	0.0036	0.036

Listed are the two non-immunoglobulin proteins detected at significantly higher levels by LC-MS/MS in pulldown samples of His-tagged spike glycoprotein mixed with plasma from COVID-19 compared to non-COVID-19 ICU patients. Significance was determined through paired t-tests with Benjamini and Hochberg's FDR correction.

Supplementary Table 9: List of primers.

Name	Description	Primer sequence (5' – 3')	Label
2019-nCoV_N1-F	N1 forward (qPCR)	GAC CCC AAA ATC AGC GAA AT	none
2019-nCoV_N1-R	N1 reverse (qPCR)	TCT GGT TAC TGC CAG TTG AAT CTG	none
2019-nCoV_N1-P	N1 probe (qPCR)	FAM-ACC CCG CAT /ZEN/ TAC GTT TGG TGG ACC-3IABkFQ	FAM, ZEN, 3IABkFQ
2019-nCoV_N2-F	N2 forward (qPCR)	TTA CAA ACA TTG GCC GCA AA	none
2019-nCoV_N2-R	N2 reverse (qPCR)	GCG CGA CAT TCC GAA GAA	none
2019-nCoV_N2-P	N2 probe (qPCR)	FAM-ACA ATT TGC /ZEN/ CCC CAG CGC TTC AG-3IABkF	FAM, ZEN, 3IABkFQ
Pseudoparticles-F	Pseudoparticles forward (PCR)	ACGCGTCGACTTTTGTGGCAAAG GTT	none
Pseudoparticles-R	Pseudoparticles reverse (PCR)	CCCAAGCTTGGGACGCGTCGTTA CGTAGAATCGAGACCGAGGAGA GGGTTAGGGATAGGCTTACCACC ACCTCCACCGCAGCATGATCCGC ATGAGC	none

Listed are the primers used for qPCR detection of the SARS-CoV-2 N1 and N2 gene and the primers used for PCR amplification during pseudoparticle production. TaqMan® probes were labeled at the 5'-end with the reporter molecule 6- carboxyfluorescein (FAM) and with a double quencher, ZEN™ Internal Quencher positioned between the ninth (9th) and tenth (10th) nucleotide base in the oligonucleotide sequence and Iowa Black® FQ (3IABkFQ) located at the 3'-end (Integrated DNA Technologies).

Supplementary Table 10: Isolation windows used in DIA-MS methods.

Precursor ion [m/z]	Isolation Window [m/z]
360.5	61
405.5	31
431, 452, 473	22
492, 509, 526, 543, 560, 577, 594, 611, 628, 645	18
664, 685, 706, 727	22
752, 781, 810, 839	30
871	36
908, 947	40
991, 1040	50
1095	62
1162.5	75

Supplementary Table 11: Features with missing data in the combined COVID-19 ICU cohorts.

Feature	Missing Count	Missing %	Imputed
Creatinine	1	1.3%	Yes
C-reactive protein	2	2.6%	Yes
Albumin	3	3.8%	Yes
Bilirubin	4	5.1%	Yes
Lymphocytes	3	3.8%	Yes
Monocytes	3	3.8%	Yes
Hemoglobin	3	3.8%	Yes
ALP (alkaline phosphatase)	3	3.8%	Yes
Respiratory rate	5	6.4%	Yes
White cell count	5	6.4%	Yes
Neutrophil	5	6.4%	Yes
SOFA score	7	9.0%	Yes
Urea	9	11.5%	Yes
ALT (alanine aminotransferase)	16	20.5%	Yes

List of features with missing data in the combined cohort i.e. GSTT and KCH cohort. Features with missing percentage less than 30% were imputed using KNN impute with K = 5. Eosinophil counts and basophil counts with missing percentage greater than 30% were not imputed and excluded from data analysis.
