# Supplementary material for: SARS-CoV-2 RNAemia and proteomic trajectories inform prognostication in COVID-19 patients admitted to intensive care
Source: Nat Commun. 2021 Jun 7;12:3406. doi: 10.1038/s41467-021-23494-1 (PMC8184784; doi:10.1038/s41467-021-23494-1)
Supplement: Supplementary file 3 — Reporting Summary [file 41467_2021_23494_MOESM3_ESM.pdf]

nature research

Corresponding author(s): Manuel Mayr
Last updated by author(s): Apr 22, 2021

Reporting Summary

Nature Research wishes to improve the reproducibility of the work that we publish. This form provides structure for consistency and transparency in reporting. For further information on Nature Research policies, see our Editorial Policies and the Editorial Policy Checklist.

Statistics

For all statistical analyses, confirm that the following items are present in the figure legend, table legend, main text, or Methods section.

n/a	Confirmed	
☐	☒	The exact sample size (*n*) for each experimental group/condition, given as a discrete number and unit of measurement
☐	☒	A statement on whether measurements were taken from distinct samples or whether the same sample was measured repeatedly
☐	☒	The statistical test(s) used AND whether they are one- or two-sided<br>*Only common tests should be described solely by name; describe more complex techniques in the Methods section.*
☐	☒	A description of all covariates tested
☐	☒	A description of any assumptions or corrections, such as tests of normality and adjustment for multiple comparisons
☐	☒	A full description of the statistical parameters including central tendency (e.g. means) or other basic estimates (e.g. regression coefficient) AND variation (e.g. standard deviation) or associated estimates of uncertainty (e.g. confidence intervals)
☐	☒	For null hypothesis testing, the test statistic (e.g. F, t, r) with confidence intervals, effect sizes, degrees of freedom and P value noted<br>*Give P values as exact values whenever suitable.*
☒	☐	For Bayesian analysis, information on the choice of priors and Markov chain Monte Carlo settings
☐	☒	For hierarchical and complex designs, identification of the appropriate level for tests and full reporting of outcomes
☐	☒	Estimates of effect sizes (e.g. Cohen's *d*, Pearson's *r*), indicating how they were calculated

Our web collection on statistics for biologists contains articles on many of the points above.

Software and code

Policy information about availability of computer code

Data collection	Proteome Discoverer software (version 2.3.0.523, Thermo Scientific) was used to search raw SARS-CoV-2 spike glycoprotein pull-down data files against a human database (UniProtKB/Swiss-Prot version 2020 01, 20,365 protein entries) supplemented with SARS-CoV-2 spike glycoprotein (1 protein entry) using Mascot (version 2.6.0, Matrix Science). The mass tolerance was set at 10 ppm for precursor ions and 0.02 Da for fragment ions. Trypsin was used as the digestion enzyme with up to two missed cleavages being allowed. Carbamidomethylation of cysteines and oxidation of methionine residues were chosen as fixed and variable modifications, respectively.
Data analysis	Statistical analysis and associated Figures were generated with R programming environment (version 4.02), Python programming environment (version 3.8.6) and GraphPad software (version 8.4.3). Schematic diagrams were created with Biorender.com. Image analyisis of syncytia and pseudoparticle entry were performed using Harmony software (version 4.9, PerkinElmer).

For manuscripts utilizing custom algorithms or software that are central to the research but not yet described in published literature, software must be made available to editors and reviewers. We strongly encourage code deposition in a community repository (e.g. GitHub). See the Nature Research guidelines for submitting code & software for further information.

Data

Policy information about availability of data

All manuscripts must include a data availability statement. This statement should provide the following information, where applicable:
- Accession codes, unique identifiers, or web links for publicly available datasets
- A list of figures that have associated raw data
- A description of any restrictions on data availability

The authors declare that the data supporting the findings of this study are available within the article and its Supplementary Data. Source data are provided with this paper. The mass spectrometry proteomics data have been deposited to the ProteomeXchange Consortium via the PRIDE partner repository with the dataset

identifiers PXD024026 and PXD024089 (https://www.ebi.ac.uk/pride/). We the following human protein database (UniProtKB/Swiss-Prot version 2020 01, 20,365 protein entries, (https://www.uniprot.org).
The external validation data was provided by the MGH Emergency Department COVID-19 Cohort (Filbin, Goldberg, Hacohen) with Olink Proteomics38 (https://info.olink.com/mgh-covid-study-overview-page?utm_campaign=Broad%2520%2520Explore%2520Covid%2520Study&utm_source=research-gate&utm_medium=MGH%2520post).

Field-specific reporting

Please select the one below that is the best fit for your research. If you are not sure, read the appropriate sections before making your selection.

- [x] Life sciences
- [ ] Behavioural & social sciences
- [ ] Ecological, evolutionary & environmental sciences

For a reference copy of the document with all sections, see nature.com/documents/nr-reporting-summary-flat.pdf

Life sciences study design

All studies must disclose on these points even when the disclosure is negative.

Sample size	-COVID-19 ICU: 295 samples from 78 patients<br>-COVID-19 non-ICU: 45 samples from 45 patients<br>-Non-COVID-19 ICU: 74 samples from 25 patients<br>-Controls: 60 samples from 30 patients<br>-Sample size was not predetermined but was powered to detect significant differences in the phenotypes of the study population.
Data exclusions	From the originally recruited 97 COVID-19 ICU patients, 19 Patients were excluded from all analyses due to missing outcome records, resulting in n=78 COVID-19 ICU patients.<br>In 3 of the 78 COVID-19 patients, only RNAemia but no proteomics were performed due to sample availability.
Replication	We used samples from patients recruited at 2 different hospitals and analyzed both plasma and serum to add robustness to the findings of our study. All findings are reported and discussed in terms of their statistical significance in our study and replication in other studies.
Randomization	Not applicable (observational cohort study)
Blinding	Not applicable (observational cohort study)

Reporting for specific materials, systems and methods

We require information from authors about some types of materials, experimental systems and methods used in many studies. Here, indicate whether each material, system or method listed is relevant to your study. If you are not sure if a list item applies to your research, read the appropriate section before selecting a response.

Materials & experimental systems

n/a	Involved in the study	
[ ]	[x]	Antibodies
[ ]	[x]	Eukaryotic cell lines
[x]	[ ]	Palaeontology and archaeology
[x]	[ ]	Animals and other organisms
[ ]	[x]	Human research participants
[x]	[ ]	Clinical data
[x]	[ ]	Dual use research of concern

Methods

n/a	Involved in the study	
[x]	[ ]	ChIP-seq
[x]	[ ]	Flow cytometry
[x]	[ ]	MRI-based neuroimaging

Antibodies

Antibodies used	ACE2 (diluted 1:1000, Abcam, ab15348), LGALS3BP (diluted 1:1000, Abcam, ab217572), SARS-CoV-2 Spike protein (diluted 1:500, GeneTex GTX632604), V5-488 (diluted 1:500, Thermo Fisher Scientific, 377500A488), α-tubulin (diluted 1:10000, Sigma-Aldrich T5168), mouse-HRP (diluted 1:10000, Abcam ab6789), rabbit-HRP (diluted 1:5000, Abcam ab205718)
Validation	Antibody validation was according to the manufacturer's information and past experience in the laboratory.

Eukaryotic cell lines

Policy information about cell lines

Cell line source(s)	HEK293T cells (ATCC CRL-3216), Vero (WHO) Clone 118 cells (ECACC 88020401)
Authentication	No cell line authentication was performed
Mycoplasma contamination	Cell lines were negative for mycoplasma contamination
Commonly misidentified lines (See ICLAC register)	None

Human research participants

Policy information about studies involving human research participants

Population characteristics	A detailed description of population characteristics is contained in the manuscript. Median (IQR) for age and % of sex are listed below:<br>-COVID-19 ICU: age: 54 (46.25, 64.01) years, sex: 71.79% male<br>-COVID-19 non-ICU: age: 60.82 (45.95, 71.58) years, sex: 66.66% male<br>-Non-COVID-19 ICU (pre-pandemic): age: 64 (38, 73), sex: 53.85% male<br>-Non-COVID-19 ICU (intra-pandemic): age: 70 (59.25, 78), sex: 7 58.33% male<br>-Controls: age: 70 (64.00, 74.75) years, sex: 73.33% male
Recruitment	COVID-19 cohorts: COVID-19-positive patients, as confirmed by RT-qPCR of nasopharyngeal samples, who were admitted to the ICUs of Guy's and St Thomas' NHS Foundation Trust (GSTT) and King's College Hospital (KCH) between March 12, 2020 and July 1, 2020, were recruited for an observational cohort study with serial blood sampling and analysis of clinical outcomes. The primary outcome measure was defined as mortality 28 days after ICU admission. Serial blood sampling was performed within 24 hours of admission to ICU and thereafter three measurements were taken during week 1, week 2 and again before discharge. In addition, we obtained plasma samples from COVID-19 patients upon hospitalization at GSTT (non-ICU COVID-19 cohort). Non-COVID-19 comparator cohorts: Plasma was collected from patients enrolled at the same time in the same KCH ICU as our COVID-19 ICU cohort but who repeatedly tested negative for nasopharyngeal SARS-CoV-2 (intra-pandemic, non-COVID-19 ICU cohort). Serial blood sampling of these samples was performed identical to our COVID-19 cohort. Additionally, pre-pandemic plasma samples from patients recruited at GSTT prior to the COVID-19 pandemic were available as controls. Firstly, this included serial plasma samples from sepsis ICU patients (pre-pandemic, non-COVID-19 ICU sepsis cohort) recruited between October 16, 2019 and February 26, 2020, collected upon admission and at three timepoints thereafter. Sepsis was defined according to Sepsis-3 definitions (infection with organ dysfunction defined using SOFA score 2 or more points). The eligibility criteria for this cohort and the study protocol have been reported before51. Secondly, plasma samples from patients before elective cardiac surgery (pre-pandemic, non-COVID-19 control cohort) recruited between July 8, 2019 and September 9, 2019.
Ethics oversight	REC19/NW/0750 for all patients recruited at KCH; REC19/SC/0187 for patients recruited at GSTT of the COVID-19 ICU cohort, the pre-pandemic sepsis ICU cohort, the pre-pandemic control cohort; REC19/SC/0232 for patients recruited at GSTT of the non-ICU COVID-19 cohort

Note that full information on the approval of the study protocol must also be provided in the manuscript.
